# Associations between preschool attendance and developmental impairments in pre-school children in a six-year retrospective survey

**DOI:** 10.1186/1471-2458-6-260

**Published:** 2006-10-20

**Authors:** Heribert L Stich, Bernhard T Baune, Riccardo N Caniato, Alexander Krämer

**Affiliations:** 1 Department of Public Health, District of Dingolfing-Landau, Obere Stadt 1, 84130 Dingolfing, Germany; 2 Department of Psychiatry and Psychotherapy, Mental Health Epidemiology, University of Muenster, 48149 Muenster, Germany; 3 Department of Psychiatry, School of Medicine, James Cook University, Australia; 4 Institute of Mental Health Services, Townsville General Hospital, Queensland, Australia; 5 Department of Public Health Medicine, School of Public Health, University of Bielefeld, 33501 Bielefeld, Germany

## Abstract

**Background:**

Many school-aged children suffer physical and mental impairments which can adversely affect their development and result in significant morbidity. A high proportion of children in western countries attend pre-school, and it is likely that the preschool environment influences the prevalence and severity of these impairments. Currently there is insufficient data available on the prevalence of these impairments and their causal associations. The influence that location of a pre-school and the duration of preschool attendance have on the prevalence of these impairments is not known.

**Methods:**

In a retrospective survey spanning six years (1997–2002) we reviewed the records of 6,230 preschool children who had undergone routine school entry assessments. These children had been assessed utilising a modified manual of the "Bavarian Model" for school entry examinations. This model outlines specific criteria for impairments of motor, cognitive, behavioural and psychosocial functioning. Prevalence rates for physical and behavioural impairments were based on the results of these assessments. The relationship between the prevalence of impairments and the duration of preschool attendance and the location of the preschool attended was estimated utilizing logistic regression models.

**Results:**

We found that 20.7% of children met the criteria for at least one type of impairment. Highest prevalence rates (11.5%) were seen for speech impairments and lowest (3.5%) for arithmetic impairments. Boys were disproportionately over represented, with 25.5% meeting the criteria for impairment, compared to 13.0% for girls. Children who had attended preschool for less than one year demonstrated higher rates of impairment (up to 19.1% for difficulties with memory, concentration or perseverance) compared to those who had attended for a longer duration (up to 11.6% for difficulties with pronouncation). Children attending preschool in an urban location had slightly elevated rates of impairment (up to 12.7%), compared to their rural counterparts (up to 11.1%).

**Conclusion:**

Our results demonstrate that there are high prevalence rates for physical and mental impairments among preschool children. Furthermore, children without preschool experience are a risk group for struggling with educational successes. The associations between the duration of preschool attendance and location of preschool attended and rates of impairment need replication and further exploration. Larger prospective studies are needed to examine if these relationships are causal and may therefore lend themselves to specific intervention strategies.

## Background

International literature seems to have increasingly focused on children's health issues over recent years [1-5] highlighting a growing understanding that intervention strategies that target children, are not only humane, but may be particularly effective in reducing the subsequent burden of disease in adults. A better understanding of the childhood developmental trajectories that lead to subsequent morbidity is essential for the implementation effective early intervention strategies. Evidence suggests that social and psychological factors may have as important an effect on child development as genetic, physical and cultural factors [6]. Some researchers have concluded that psychosocial factors are more important than biological ones in influencing the development of children [7].

Risk factors for poor health outcomes in children, such as lower socio-economic class, housing problems, psychological diseases of parents and poor social integration of families, have been investigated by previous authors [8]. There appears to be relatively little research, however, focused on identifying factors of attendance of a preschool associated with child development.

Results of previous studies suggests that physical and psychological disorders in children occur when risk factors outweigh protective factors during critical childhood stages, especially during the pre-school period of development [9,10]. Studies have shown that boys are at a significantly greater risk of developing psychological disorders during childhood than girls [11-13]. There is limited research on how specific factors, such as the preschool environment, for instance, modulate the prevalence of significant impairments. Some authors have suggested that the pre-school milieu is as important as the family environment for healthy child development [14-16]. Children learn different and essential practical skills and social competences in the pre-school setting of a preschool, which are likely to impact significantly on subsequent functioning. In this epidemiological study we investigate the effect that location of a preschool and duration of preschool attendance has on the physical, psychological and behavioural well-being of pre-school children.

## Methods

We analysed all the school entry assessment records (N = 6,420) of the German district of Dingolfing-Landau for six consecutive years (1997–2002). School regulations in the province of Bavaria mandate that all children undergo a developmental assessment and a standardized physical examination before entering primary school [17,18]. The examinations consist of a modified version of the "Bavarian Model" for school entry examinations [19], which are carried out by a specially trained physician. These examinations assess four main developmental areas: motor, speech, cognitive and psychosocial development. Corresponding to these areas of development a standardized medical examination was carried out (Tab 1). The medical examination is manualized and standardized, and was carried out by the same qualified medical specialist over the whole study period. The medical examiner was not part of the study team and was blind to the hypotheses of the study. Social-epidemiological data for every child, such as age, gender, nationality, number of siblings, duration and location of a preschool attendance were collected from the parents.

Impairments of motor development were documented if the child had difficulties with two or more the standardised tests. Impairments of speech, cognition and psychosocial development were diagnosed if a child was unable to complete at least one of the standardised medical tests correctly (Tab. 1).

### Statistics

Prevalence rates of impairments were stratified according to age, gender, duration of preschool attendance (< 1 year vs. ≥1 year) and location (urban vs. rural) of the preschool attended. Preschools were defined as rural in areas of less than 10,000 inhabitants and as urban where the documented population was over 10,000. The duration of attendance at preschool was classified dichotomously as either less or more than one year in total duration. Associations between duration of attendance and location of the preschool, and developmental impairments, were calculated utilising logistical regression models, which were adjusted for age, gender and nationality. Nationality was grouped into either German or non-German. All statistics were carried out by the use of the Software package SPSS Version 12.0.

## Results

### Target population

During school entry examinations 6,420 children with an average age of 6.04 years (SD+/-0.37) were investigated. There was a slight overrepresentation of boys in the sample, with 51.7 % (3,321/6,420) of the children being male and 48.3% female (3,099/6,420). The average age of girls was 6.02 years (SD+/-0.37) and of boys it was 6.05 years (SD+/-0.36). Most of the children were German nationals (93.8%). We obtained incomplete data sets of 190 children. Incomplete data sets resulted from unclear or ambiguous medical assessments, from changing locations and from examinations being carried out by doctors in private practices without reporting the outcomes to the Public Health Services. The final sample entered into the analysis was 6,230 children.

### Duration of attendance and locality of preschool

Most of the children had attended preschool for some period (92.9%). Children who had no documented preschool attendance (7.1% of the sample), were not included in the analysis. Of the children who had attended preschool, 98.3% had attended for over one year while and only 1.7% had attended for less than one year. The location of the preschool attended was recorded in 99.7% of the children. Significantly more children attended preschool in rural (73%) rather than urban (27%) locations.

### Prevalence rates of impairments

#### Motor development

Impairments of fine motor coordination (6.6%) were more common than impairments of gross motor skills (5.9%). Grapho-motor coordination, an assessment of the ability to draw simple figures, was impaired in 4.3% of the sample. Children younger than six years of age demonstrated higher rates of motor impairments than their older peers. Boys were almost three times as likely to demonstrate motor impairments than girls (Tab. 2). Children who visited a preschool for less than 1 year were also more likely to demonstrate deficits of motor development. No differences in motor development were seen between children having attended either rural or urban preschools (Tab. 3).

#### Development of speech

Three separate areas of speech were assessed: pronunciation, grammar and rhythm of speech. Abnormalities of pronunciation were present in 11.5% of the children, while deficits in grammar (2.9%) or rhythm (3.3%) were far less common. Boys were over twice as likely to demonstrate deficits in speech development, than girls (Tab. 2). No significant differences in speech development were observed after stratifying for age, duration and location of a preschool attendance (Tab. 3).

#### Development of cognition

The most commonly observed impairments were of memory and concentration (8.4%), while the lowest prevalence rate was found for deficits of arithmetic abilities (3.5%). In general, we observed that boys showed twice the rate of cognitive deficits of girls. Children younger than six years of age had twice the rate of cognitive problems than their older peers (Tab. 2). Cognitive deficits were 3–4 times as prevalent in children who had attended preschool for less than 1 year. There was no difference in the rates of cognitive impairment between children attending urban or rural preschool settings (Tab. 3).

#### Psychosocial development

The psychosocial abilities were judged to be impaired in 5.9% of the study population. Children under six years of age were twice as likely to demonstrate psychosocial deficits than older children. Boys demonstrated higher rates of psychosocial problems (7.0%) than girls (4.7%). Duration of preschool attendance and location of the preschool attended did not affect psychosocial development.

#### Associations between impairments and duration of attendance and location of preschool

Children who had attended preschool for less than one year had significantly higher rates of impairment in most areas of psychosocial functioning. Children attending preschool for less than one year were four times as likely to demonstrate impairments of fine and gross motor skills (Tab. 4). Impairments in cognition, memory and concentration were also significantly more prevalent in children who had attended preschool for the briefer period. In the area of speech, however, the children who had attended preschool for less than one year actually demonstrated lower rates of impairment.

In all areas of development assessed, rural preschool children demonstrated lower rate of impairments than their urban counterparts (Tab. 4). The differences, however, were reasonably small and certainly not as pronounced as the differences between the children who had attended preschool for less or more than one year.

## Discussion

In a retrospective survey in the Lower Bavarian district (Germany) the prevalence rates for motor, speech, cognitive and psychosocial impairments in six consecutive cohorts of school beginners were estimated. The relationship between these impairments and the duration of preschool attendance and the location of the preschool attended were assessed. Attending preschool for less than one year was a significant risk factor for several types of impairments while children attending an urban preschool had an increased risk of impairment relative to their rural counterparts. Since our data comes from a circumscribed geographical area, it may not be equally applicable to other national or international localities.

### Prevalence rates of impairments

In our study about 2.8% girls and 7.3% boys demonstrated one or more motor impairments. Higher rates of impairment of motor development were seen in a previous study of school children in the district Weser-Ems [20]. In this study 6.6% of girls and 12.5% of boys demonstrated deficits of gross motor coordination and 6.2% girls and 17.6% boys had deficits of fine motor coordination. High rates of motor impairments were also found in children in Western German District of Northrhine-Westfalia [21]. In this study about 5.2% (2.191) girls and 11.1% (5.009) boys were assessed as having of problems of motor coordination. Impairment of fine motor coordination was found in 5.6% girls and 4.8% boys in a study of the District of Lower Saxonia (Germany) [22]. Our study, therefore, demonstrated somewhat lower rates of motor abnormalities compared to these other reports. These differences are likely to represent slight differences in school entry examinations and diagnostic procedures rather than differing prevalence rates between these populations, although the possibility of true regional differences cannot be excluded.

In the area of speech development we found that 11.5% children (6.9% girls; 15.1% boys) demonstrated impairment in pronunciation, 2.9% (1.6% girls, 3.9% boys) had impairments in grammar and 3.3% (1.9% girls; 4.4% boys) demonstrated impairments in rhythm of speech. Other reports on children's health have estimated higher rates of speech impairments than our study. A report from the district of Weser Ems for school entry children detected speech impairments in 36.2% girls and 63.8% boys [20].

To our knowledge, cognition has not been assessed in similar reviews of school entry children. The assessments utilised in our survey involved the systematic categorisation of cognitive deficits into five subgroups. We found no similar or comparable assessments documented in any previous pre-school cohorts. The large numbers of children assessed in our study and the fact that the assessments where carried out by the same medical team blind to the analysis over the study period of six years, adds to the reliability and validity to our findings. Our study indicates significant levels of cognitive impairments in the children assessed. Unfortunately, no data on cognition is available for the comparison of prevalence rates to those of other methodologies. Further studies in this field are required to obtain results for comparisons.

In our study psychosocial deficits were observed in 5.9% children (4.5% girls; 6.8% boys). Lower rates of psychosocial deficits were found in a cohort of school entry children in Northrhine-Westfalia, in 1999 (2.9% girls; 4.6% boys) and in the following year (3.0% for girls; 4.9% for boys) [23]. In contrast to these results, a study in Lower Saxonia detected significant behavioural deficits in 35.5% girls and 64.5% boys [24]. It is likely that the criteria utilised to diagnose psychosocial deficits, are not sufficiently homogenous and validated to allow the comparison of prevalence rates between different studies.

### Associations between duration and location of preschool and children's health

In our study, 91.7% of the children beginning school between 1997 and 2002 had visited a preschool before entering the first class of school. Of these children, 98.3% had attended preschool more than one year and while only a small minority of children (1.7%) had attended for less than twelve months. A general trend over time, in our study, was that the proportion of children attending preschool for less than one year declined between 1997 (2.3%) and 1999 (0.9%). In a survey of Lower Saxonia, children entering school had attended preschool for between 2 to 3 years in urban areas and between 1–2 years in rural areas [24]. According to a health report from the city of Mannheim only 2.3% of children had not visited a preschool before entering school [25]. All other children (97.7%) in Mannheim had visited preschool on average for an average of 37 months [25]. In marked contrast to these figures, a survey of children in urban Cologne found only 63% of 10,086 children entering school had visited a preschool [26].

While previous public health reports on children's health have assessed the duration of preschool attendance for children entering school, they have not attempted to assess the relationship between attendance and the prevalence of developmental impairments [22,24,25]. We identified two other small studies which investigated the relationship between duration and location of attending a preschool and the health of pre-school children [20,28]. Similar to our study, a survey in Brandenburg, Germany, [28] reported a four-fold risk for impairments in fine and grapho-motor coordination for children who had visited preschool for less than one year. A study by Bruns-Philipps concluded that children who had never visited preschool suffered higher rates of deficits in fine motor coordination than children who had visited for longer than three years [20]. Our study also demonstrated higher rates of cognitive problems in children who had attended preschool for less than a year, however, there were no significant difference between the groups in psychosocial development. Somewhat surprisingly, for impairments of speech it was the children who had attended preschool for less than a year who had slightly lower rates of impairment.

The positive effects of attending a preschool for a longer period might be explained by various educational, psychological and social factors. In general, the preschool setting encourages children to utilise and model socially appropriate behaviour and supports the development of various skills and competencies. The socialisation process in a preschool setting may help foster healthy child development. Socially disadvantaged children are far less likely to attend a preschool [28], yet are also at higher risk of suffering from developmental impairments. Since in our study children who had never attended preschool, 7.1% of the initial cohort, were excluded form the analysis, we may have underestimated rates of impairment. In our study only 1.7% of children had attended preschool for less than a year. Such a small sample size reduces the reliability of our findings. While our study found an association between duration of preschool attendance, we cannot be certain that this is a causal relationship due to the cross-sectional design of the study. For instance, it is conceivable that parents of more impaired children delay their entry into preschool. Shorter preschool attendance might represent a marker for an impaired state that prevents children from being enrolled. In practice, Public Health services, which are responsible for the enrollment of children in primary school and are accountable for the medical examination of all pre-school aged children, might be able to promote more accessibility of quality preschool experiences to children with social disadvantage through improving and emphasizing the recruitment process of children and parents from socially disadvantaged areas. More studies, especially those focusing on socially disadvantaged children are needed, in order to determine causative associations.

The location of a preschool seems to have a clear though less marked influence on impairments, when compared to duration of preschool attendance. Visiting a preschool in an urban region was associated with a higher risk for all the developmental deficits which we assessed. Of note is in our study rural children were significantly over represented, and that this may result in certain selection biases. To our knowledge, no previous studies have assessed the association between children's development and location of a preschool. We therefore have no other epidemiological data against which to compare our findings.

Other factors than longer duration and rural location of a preschool, so called protective factors, optimizing children's development and health are related to healthy eating, sports, social/family support and oral/dental hygiene [29]. Preventive strategies targeting at young children can be implemented at an individual, community or national level.

## Conclusion

In our study we found relatively high rates of developmental impairments of pre-school children. Boys had significantly higher rates of impairment in most areas of development. These impairments might lead to significant morbidity in subsequent years, and have important implications for the planning and implementation of preventative health measures. Our findings support the conclusions that children without preschool experience are a risk group for struggling with educational successes and visiting a preschool may improve the physical, cognitive and psychological development of children. The observation of a cross-sectional relationship between the duration of visiting a preschool and impairment needs replication in further, including prospective studies looking at larger proportions of children not attending a preschool.

## Competing interests

The author(s) declare that they have no competing interests.

## Authors' contributions

HLS has made substantial contributions to conception and design, has examined the children, analyzed and interpreted data and drafted the manuscript.

BTB has made substantial contributions to conception and design, analysed and interpreted data, has been involved in drafting the manuscript and revising it critically for important intellectual content.

RNC has made substantial contributions to the writing of the manuscript.

AK has been involved in drafting the manuscript or revising it critically for important intellectual content and has given final approval of the version to be published.

All authors read and approved the final manuscript.

## Pre-publication history

The pre-publication history for this paper can be accessed here:



## Figures and Tables

**Table 1:** Areas of child development and corresponding standardised tests

**main areas of development**	**subdivision of development**	**standardised tests**
motor development	gross motor skills	standing on one leg; jumping on one leg; going like a rope dancer; going with clapping hands
	fine motor coordination grapho- motor coordination	finger-opposition-test; drawing different figures; drawing of a person
development of speech	pronunciation	repeating words
	grammar	retelling a short story; retelling a short picture story; explaining rules of a well known game
	rhythm of speech	repeating sentences
development of cognition	memory and concentration	repeating sentences with 7–10 words including three adjectives; repeating four single numbers in the correct sequence
	perseverance	discontinuity of capacity during the whole time of medical examinations
	abstraction	building pairs; finding out a object of various objects belonging together
	visual perception	reception and knowing of simple geometric figures or silhouettes of figures and animals
	arithmetic	counting from 1 to 10 in the correct sequence
psychosocial development		behaviour, emotionality, psycho motor et cetera

**Table 2:** Prevalence rates of impairments by age and gender

**Impairments of development**	**Age**		**Gender**	
	
	**under 6 years N = 3,084 % **(m = 83)	**6 years and older N = 3,146 % **(m = 109)	**Girl N = 2,984 % **(m = 93)	**Boy N = 3,246 % **(m = 99)
gross motor skills	7.6	4.3	3.2	8.3
fine motor coordination	9.0	4.5	3.2	9.8
grapho-motor coordination	5.4	3.4	2.1	6.2
pronunciation	11.5	11.3	7.2	15.1
grammar	3.2	2.7	1.7	4.0
rhythm of speech	3.5	3.1	1.9	4.5
memory and concentration	12.3	5.2	6.4	10.4
perseverance	8.0	2.6	4.2	6.0
abstraction	4.9	2.6	2.4	4.9
visual perception	6.0	3.9	2.9	6.7
arithmetic	4.5	2.7	2.8	4.2
psychosocial development	8.4	3.7	4.7	7.0

m: missing cases

**Table 3:** Prevalence rates of impairments by duration and location of the preschool

**impairments**	**duration**		**location**	
	
	**under 1 year N = 106 **(m = 6)	**1 year and longer N = 6,124 **(m = 27)	**Rural N = 4,548 **(m = 103)	**Urban N = 1,682 **(m=87)
gross motor skills	8.5	5.8	5.0	8.2
fine motor coordination	18.1	6.3	6.1	7.9
grapho-motor coordination	14.9	4.0	4.1	4.8
pronunciation	6.4	11.6	11.1	12.7
grammar	2.1	2.9	2.3	4.7
rhythm of speech	1.1	3.3	2.8	4.4
memory and concentration	19.1	8.0	7.6	10.8
perseverance	19.1	4.7	4.5	6.8
abstraction	13.8	3.3	3.0	5.7
visual perception	19.1	4.4	3.9	7.7
arithmetic	11.7	3.1	2.7	5.7
psychosocial development	6.4	5.8	5.4	7.2

m: missing cases

**Table 4:** Associations of impairments of development and time of visiting a preschool*/location of preschool*

**impairments of development**	**visiting a preschool shorter than 1 year**			**urban location of preschool**		
	reference (≥1 year)	Odds Ratio	95% CI**	reference (rural)	Odds Ratio	95% CI**

gross motor skills	1	1.47	0.69 – 3.13	1	**1.63**	**1.3 – 2.05**
fine motor coordination	1	**3.95**	**2.22 – 7.03**	1	**1.23**	**1.01 – 1.60**
grapho-motor coordination	1	**4.60**	**2.45 – 8.51**	1	1.09	0.82 – 1.45
pronunciation	1	0.6	0.26 – 1.40	1	**1.2**	**1.0 – 1.43**
grammar	1	0.78	0.19 – 3.25	1	**2.17**	**1.6 – 2.95**
rhythm of speech	1	0.40	0.06 – 2.91	1	**1.65**	**1.23 – 2.23**
memory and concentration	1	**2.32**	**1.33 – 4.03**	1	**1.27**	**1.04 – 1.55**
perseverance	1	**4.66**	**2.65 – 8.18**	1	**1.4**	**1.09 – 1.79**
abstraction	1	**3.37**	**1.77 – 3.42**	1	**1.56**	**1.2 – 2.1**
visual perception	1	**3.88**	**2.14 – 6.70**	1	**1.65**	**1.28 – 2.12**
arithmetic	1	**3.13**	**1.58 – 6.18**	1	**1.77**	**1.33 – 2.37**
psychosocial development	1	1.07	0.46 – 2.50	1	**1.31**	**1.04 – 1.66**

* multiple logistic regression models adjusted for age, gender and nationality

** 95% CI: 95% confidence interval
